# Cold plasma selectivity and the possibility of a paradigm shift in cancer therapy

**DOI:** 10.1038/bjc.2011.386

**Published:** 2011-10-06

**Authors:** M Keidar, R Walk, A Shashurin, P Srinivasan, A Sandler, S Dasgupta, R Ravi, R Guerrero-Preston, B Trink

**Affiliations:** 1 Mechanical & Aerospace Engineering Department, George Washington University, Phillips Hall 723, Washington, DC 20052, USA; 2 Applied Plasma Science, LLC, Baltimore, MD, USA; 3 Children's National Medical Center, Washington DC, USA; 4 Department of Human and Molecular Genetics, VCU Institute of Molecular Medicine, VCU Massey Cancer Center, Virginia Commonwealth University, School of Medicine, Richmond, VA, USA; 5 Department of Otolaryngology, Head and Neck Cancer Research Division, Johns Hopkins University, School of Medicine, Baltimore MD, USA

**Keywords:** cold plasma, cancer treatment, tumour ablation

## Abstract

**Background:**

Plasma is an ionised gas that is typically generated in high-temperature laboratory conditions. However, recent progress in atmospheric plasmas has led to the creation of cold plasmas with ion temperature close to room temperature.

**Methods:**

Both *in-vitro* and *in-vivo* studies revealed that cold plasmas selectively kill cancer cells.

**Results:**

We show that: (a) cold plasma application selectively eradicates cancer cells *in vitro* without damaging normal cells; and (b) significantly reduces tumour size *in vivo*. It is shown that reactive oxygen species metabolism and oxidative stress responsive genes are deregulated.

**Conclusion:**

The development of cold plasma tumour ablation has the potential of shifting the current paradigm of cancer treatment and enabling the transformation of cancer treatment technologies by utilisation of another state of matter.

Plasma is an ionised gas that is typically generated in high-temperature laboratory conditions. Recent progress in atmospheric plasmas has led to the creation of cold plasmas with ion temperature close to room temperature (Fridman, 2008; Fridman *et al*, 2008). Earlier studies demonstrated the non-aggressive nature of the cold plasma (Stoffels *et al*, 2002). After it was shown, albeit indirectly, that plasma can interact with organic materials without causing thermal/electric damage to the cell surface, several biological applications were examined (Stoffels *et al*, 2002, 2008). As evidence accumulates, it is becoming clear that low-temperature or cold plasmas have an increasing role to take part in biomedical applications. The potential use in biomedical applications has driven the development of a variety of reliable and user-friendly plasma sources (Laroussi and Lu, 2005; Becker *et al*, 2006; Stoffels *et al*, 2006; Fridman *et al*, 2008; Kong *et al*, 2009; Morfill *et al*, 2009). There is still some controversy with respect to the mechanism of plasma–cell interaction. Some authors are of the opinion that ion species have the most important role in plasma–cell interactions by triggering intracellular biochemistry (Fridman *et al*, 2009). Alternatively, others have suggested that neutral species have the primary role in some plasma–cell interaction pathways (Kalghatgi *et al*, 2009). Furthermore, the effects of various ion species may be highly selective; different species can have either ‘plasma-killing’ (such as O) or ‘plasma-healing’ (such as NO) effects (Fridman *et al*, 2008; Kong, 2009). The role of other species, such as O_3_ and OH, are not yet clear.

Even less clear is the nature of the interaction between cold plasmas and cancer tissue. Only limited research into the utility of cold plasma for cancer therapy has been performed. For the most part, these *in vitro* studies are limited to skin cells and simple cellular responses to the cold plasma treatment (Kim *et al*, 2010; Zirnheld *et al*, 2010; Georgescu and Lupu, 2010). In addition, preliminary reports on plasma's *in-vivo* antitumour effect are reported (Vandamme *et al*, 2010). Recent studies have delineated the effects of cold plasma on both the cellular and sub-cellular levels. On the cellular level, plasma effects include detachment of cells from the extracellular matrix and decreased migration velocity of cells. On the sub-cellular level, cell surface integrin expression is reduced (Shashurin *et al*, 2008, 2010). In this study, we examined the therapeutic potential of a cold plasma jet in cancer cell lines and tumours, focusing on selective tumour cell eradication capabilities and signalling pathway deregulation.

## Materials and methods

### Cold plasma source

The cold plasma source developed at GWU (Shashurin *et al*, 2008, 2010) is equipped with a pair of high-voltage (HV) electrodes, a central electrode and an outer ring electrode. Electrodes are connected to a secondary coil of HV resonant transformer operating at a voltage of about 2–5 kV and a frequency of about 30 kHz, with a helium flow rate of about 11 l min^−1^. The visible plasma jet had a length of approximately 5 cm and was well collimated along the entire length. According to previous studies (Shashurin *et al*, 2008, 2010), the plasma jet is discontinuous and represents a series of propagating plasma bullets.

### *In-vitro* assays

*Lung cancer cell lines* We examined the normal human Bronchial epithelial (NHBE) and the lung cancer (SW900) cell lines. Cold plasma treatments were carried out at HV in the range of 3–5 kV, helium flow in the range 10–20 l min^−1^, distance from plasma source to cells of about 1 cm and treatment durations of about 30 s (Figure 3A).

*Murine melanoma cells* B16-F10 melanoma cells were purchased from American Type Culture Collection (Manassas, VA, USA). Cells were cultured in D10 media (Dulbecco’s modified eagle’s medium (DMEM) containing 10% fetal calf serum, 1% penicillin/streptomycin and 1% L-glutamine).

*Primary macrophage harvest and growth* Primary bone marrow macrophages were directly harvested from the tibias and femurs of killed mice. Cells were cultured in 30% L929 cell-conditioned media, re-fed on either day 2 or 3, and plated between days 6 and 9.

*Flow Cytometry* Triplicate samples of 1 × 10^4^ murine macrophages and B16-F10 melanoma cells were plated into 96-well plates with 100 *μ*l of D10 media (DMEM (Sigma-Aldrich, St Louis, MO, USA)), supplemented with 10% fetal bovine serum, penicillin (100 IU ml^−1^), streptomycin (100 *μ*g ml^−1^; Sigma-Aldrich) and 1% L-Glutamine (Mediatech Inc., Manassas, VA, USA)) per well. After cold plasma treatment, cells were collected and stained with fluorescein isothiocyanate-conjugated Annexin V and 7-aminoactinomycin D (7-AAD) obtained from BD Biosciences (San Jose, CA, USA). Flow cytometry was performed using FACS Calibur (BD Bioscience); results were analysed using FlowJo software (Ashland, OR, USA).

### *In-vivo* assays

B16 and subcutaneous bladder cancer tumours (SCaBER) cells of 2 × 10^5^ were subcutaneously injected into the right hind legs of 8 C57Bl6 mice (four control and four treated) and 10 nude mice (five control and five treated), respectively. B16 tumours were treated with cold plasma once they were approximately 5 mm in maximum diameter. Control mice received no therapy after inoculation. All treated mice received 5 min of cold plasma treatment. Tumours were treated through the skin; no overlying incisions were made. Mice received one round of treatment only. Tumour volumes were calculated using the formula V=0.52(X^2^Y). Control and treatment mice were killed when tumours reached a maximum diameter of 20 mm, if tumour bleeding or ulceration occurred, or if the mice appeared moribund.

The cold plasma jet was also applied to nude mice bearing SCaBER. We examined the mouse skin after 2–5-min cold plasma treatment, to compare gross tissue damage to the skin before and after treatment. We extracted RNA to perform gene expression analyses.

### Gene expression assays

A gene expression profile of treated and untreated tumour was obtained using the genome-wide HumanHT-12 v4 Expression BeadChip arrays (Illumina, San Diego, CA, USA). Each array on the HumanHT-12 Expression BeadChip targets more than 25 000 annotated genes, with more than 48 000 probes derived from the National Centre for Biotechnology Information Reference Sequence (NCBI RefSeq; Build 36.2, Rel 22) and the UniGene (Build 199) databases.

Total RNA was prepared as described in the RNeasy Mini Kit (Qiagen, Germantown, MD, USA) with on-column DNase I digestion. All samples were processed at the Sidney Kimmel Comprehensive Cancer Centre Microarray Core Facility at Johns Hopkins University (Baltimore, MD, USA). Briefly, 500 ng total RNA from each sample was amplified and labelled using the Illumina TotalPrep RNA Amplification Kit, AMIL1791 (Ambion, Austin, TX, USA) as described in the instruction manual. All arrays were hybridised at 58 °C for 16–20 h, followed by wash and stain procedures according to the Whole-Genome Gene Expression Direct Hybridization Assay Guide (Illumina). Fluorescent signals were obtained by scanning with iScan System, and data were extracted with Gene Expression Module 1.0.6 in GenomeStudio 1.0.2 (Illumina) with or without background subtraction.

*Ingenuity pathway analysis (IPA)* Pathway and ontology analysis were performed to identify how the observed expression changes between treated and untreated tumour tissue alter cellular networks and signalling pathways. A list of RefSeq identifiers for up/downregulated genes was uploaded to the IPA Programme (Ingenuity Systems, Redwood City, CA, USA), enabling exploration of gene ontology and molecular interaction. Each uploaded gene identifier was mapped to its corresponding gene object (focus genes) in the Ingenuity Pathways Knowledge Base. Core networks were constructed for both direct and indirect interactions using default parameters, and the focus genes with the highest connectivity to other focus genes were selected as seed elements for network generation. New focus genes with high specific connectivity (overlap between the initialised network and immediate connections of the gene) were added to the growing network until the network reached a default size of 35 nodes. Non-focus genes (those that were not among our differentially expressed input list) that contained a maximum number of links to the growing network were also incorporated. The ranking score for each network was then computed by a right-tailed Fisher's exact test as the negative log of the probability that the number of focus genes in the network is not due to random chance. Similarly, significances for functional enrichment of specific genes were also determined by the right-tailed Fisher's exact test, using all input genes as a reference set.

*Statistical analysis* Results are representative of three independent experiments. Error bars represent s.e.m. Statistical analysis was performed using GraphPad Prism 5 Software (GraphPad Software, Inc., La Jolla, CA, USA). For *in-vitro* assays, one-way ANOVA with Bonferroni's post-test was performed to determine the differences in viable cells, both between all groups and between treatment groups and controls. For *in-vivo* survival, Kaplan–Meier curves were developed and Log-rank (Mantel–Cox) testing was performed. For gene analyses, the ranking score for each network was computed by a right-tailed Fisher's exact test as the negative log of the probability that the number of focus genes in the network is not due to random chance. Similarly, significances for functional enrichment of specific genes were also determined by the right-tailed Fisher's exact test, using all input genes as a reference set.

*Ethical statement* For the proposed studies, we utilised procedures detailed in a protocol approved by the Johns Hopkins Animal Care and Use Committee (Protocol no. MO09M47) and Children's National Medical Centre ACU committee (Protocol no. 198-12-06). All animals were subjected to strict supervision and veterinary care by the Division of Comparative Medicine at the Johns Hopkins University School of Medicine and the Research Animal Facility at the Children's National Medical Centre. Animal care complied with Federal and State regulations regarding proper and humane treatment of animals.

## Results

### *In-vitro* cold plasma treatment to cell lines

**Figure 1:**
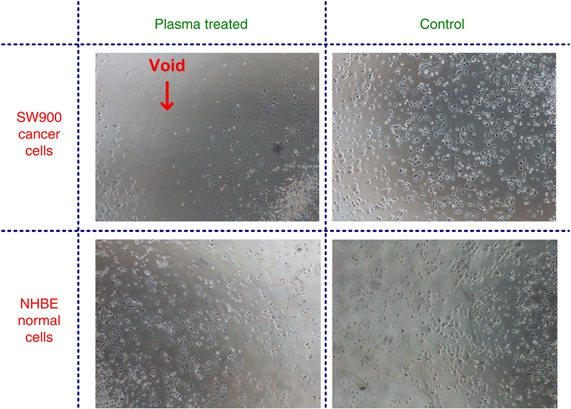
Selectivity effect of plasma treatment: SW900 cancer cells were detached from the plate in the zone treated with plasma, whereas no detachment was observed in the treated zone for the normal NHBE cells.

A strong selective effect was observed; the resulting 60–70% of SW900 cancer cells were detached from the plate in the zone treated with plasma, whereas no detachment was observed in the treated zone for the normal NHBE cells under the same treatment conditions. Images of treated and untreated NHBE and SW900 cells are shown in Figure 1. Plasma treatment leads to a significant reduction in SW900 cell count, whereas NHBE cell count is practically unchanged. Both murine macrophages and B16 melanoma cells were treated with the cold plasma device for 0, 30, 60 and 120 s. Annexin V and 7-AAD staining was performed for flow cytometry analysis at 24 and 48 h after treatment.

**Figure 2:**
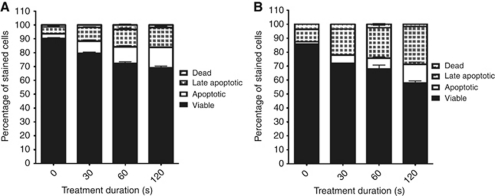
Selectivity effect of plasma treatment: B16 melanoma cells treated with the cold plasma device for 0, 30, 60 and 120 s. (**A**) 24 h; (**B**) 48 h. Annexin V and 7-AAD staining was performed for flow cytometry analysis at 24 and 48 h after treatment. Four-quadrant analysis of the results characterises the cells as viable (unstained), apoptotic (Annexin V positive), late-apoptotic (double positive) and dead (7-AAD positive).

As seen in Figure 2, a clear dose response to cold plasma treatment is seen in the murine melanoma cells at both 24 and 48 h (*P*<0.0001), whereas the treated murine macrophages do not differ from control at either 24 or 48 h (*P*=0.1350 and 0.1630, respectively). These findings suggest that the cold plasma jet has a more selective effect on murine melanoma cells.

### *In-vivo* studies

**Figure 3:**
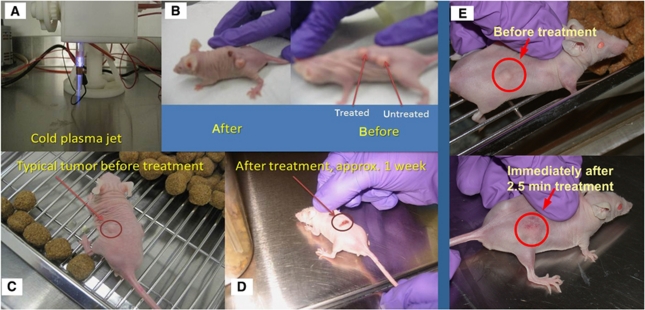
(**A**) Cold plasma device; (**B**) typical image of mice with three tumours before and after treatment (shown after 24 h); (**C** and **D**) typical image of mice with a single tumour before and approximately 1 week after treatment; (**E**) tumour before and immediately after the 2.5-min treatment with cold plasma jet.

To determine the cold plasma effect *in-vivo*, we applied the cold plasma jet to nude mice bearing SCaBER. We examined the mouse skin after cold plasma treatment and did not see any damage to the skin after 2–5 min of treatment. Tumour models treated by cold plasma are shown in Figure 3. The plasma jet is shown in Figure 3A. One can see that a single plasma treatment leads to tumour ablation with neighbouring tumours unaffected (see Figure 3B). These experiments were performed on 10 mice with the same outcome. We found that tumours of about 5 mm in diameter are ablated after 2 min of single time plasma treatment (see Figure 3B and E), whereas larger tumours decreased in size. Interestingly, ablated tumours did not grow back, whereas partially affected tumours started growing back a week after treatment, although they did not reach the original size even after a 3 weeks aftertreatment (Figure 3C and D).

**Figure 4:**
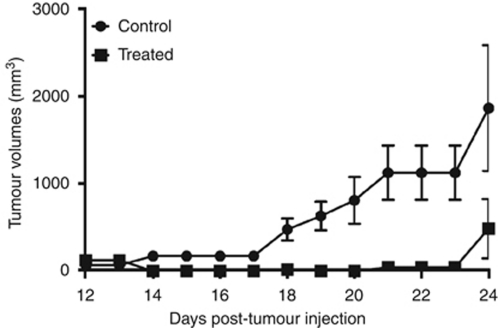
Cold plasma treatment effect on the growth of established tumour in a murine melanoma model.

**Figure 5:**
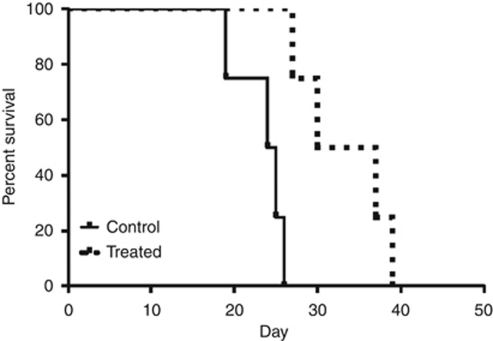
Cold plasma treatment effect on the mice survival in a murine melanoma model.

We next evaluated the cold plasma device for *in-vivo* efficacy in a murine melanoma model. Although tumours eventually recurred, a single transcutaneous cold plasma treatment induced ablation of the tumour through the overlying skin. As demonstrated in Figure 4, tumour growth rates were markedly decreased after cold plasma treatment. Notably, this resulted in a markedly improved survival in the treatment group (*P*=0.0067), with a median survival of 33.5 *vs* 24.5 days as shown in Figure 5.

### Skin temperature is not increased by cold plasma treatment

The skin temperature during plasma treatment was measured using an infra-red thermometer (Traceable, Model no. 4470, Control Company, Friendswood, TX, USA) to assess whether the cold plasma effect on cancer tissue is associated with thermal damage. Cold plasma treatment produced an increase in skin temperature of approximately 2 °C above room temperature, which is below the temperature needed for thermal damage.

### Gene expression analysis of cold plasma-treated bladder cancer tumour tissue demonstrates alteration in various pathways

**Figure 6:**
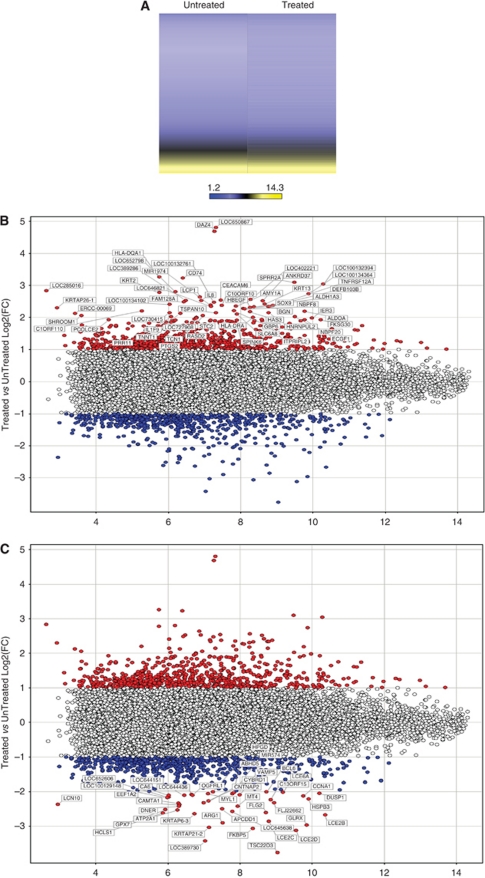
(**A**) A heatmap of the normalised log2 signal intensity values in the Illumina expression array for the untreated and treated sample. The colour yellow was selected to represent high log2 signal intensity values and the colour blue to represent low log2 signal intensity values. (**B**) MvA plot of upregulated genes (red) in a treated sample compared to an untreated sample. The values on the Y axis represent the ratio of treated/untreated log2 Fold Change. The values on the X axis represent the average log2 signal intensity. (**C**) MvA plot of downregulated genes (blue) in a treated sample compared to an untreated sample. The values on the Y axis represent the ratio of treated/untreated log2 Fold Change. The values on the X axis represent the average log2 signal intensity.

The beta values of all probes on the Illumina BeadChip arrays were subjected to log10 transformation and then normalised to the average to generate a heatmap of selected genes on the basis of unsupervised hierarchical clustering with the Spotfire software (TIBCO, Somerville, MA, USA). The clustering was based on the unweighted average method using correlation as the similarity measure and ordering by average values. The colour red was selected to represent upregulated genes and the colour green to represent downregulated genes. Genes were selected for clustering if they were four times upregulated or downregulated after treatment with cold plasma. Figure 6 depicts the most upregulated genes (left panel) and the most downregulated genes (right panel) after cold plasma treatments. Supplementary Table 1 lists the genes that were differentially expressed after cold plasma treatment.

Differences between genes that were up- or downregulated in treated or untreated cells were analysed for biological significance using Geneontology (Spotfire) and IPA. Differences in gene expression were found to be associated with pathways intimately related with cell adhesion, cell proliferation, growth regulation and cell death (*P*<0.05).

The top associated network functions in IPA, shown in Supplementary Table 2, are pathways directly related to organismal injury and abnormalities: cellular development, cell signalling, cellular movement, dermatological diseases and conditions, cell death, and inflammatory response (*P*<0.05).

Several genes associated with the apoptotic and oxidative stress pathways were deregulated in tumours treated with cold plasma. Details of the upregulated (Supplementary Figure 1) and downregulated (Supplementary Figure 2) networks are presented in the Supplementary section.

## Discussion

Our experiments demonstrate potent effects of cold plasma treatment on cancerous tissue both *in vitro* and *in vivo*. Previous research has offered several potential mechanisms for cold plasma's effect, including development of reactive oxygen species (ROS), reactive nitrogen species (RNS), charged particles, heat, pressure gradients, and electrostatic and electromagnetic fields (Furchgott, 1999). Notably, plasma has minimal impact on ambient cellular conditions. For instance, media pH levels remain unchanged after treatment (Volotskova *et al*, 2010), and our study confirms that thermal effects associated with cold plasma are negligible. Beyond the direct external influence of the jet, cold plasma may induce living cells to produce their own ROS/RNS. Thus, these preliminary results suggest that multiple pathways involved in cancer processes, including cell adhesion, cell proliferation, growth regulation and cell death, are selectively deregulated by cold plasma treatment in cancer cells. Some of these pathways may likely be responsible for tumour ablation. Perhaps consequently, we further demonstrate induction of cellular apoptosis in treated cells, as manifested by both expression of cell-surface markers and gene expression, confirming results of previous studies (Kieft *et al*, 2006). Most importantly, these findings are translated to *in vivo* models of cancer therapy, with marked reductions in tumour volumes and improved survival.

Given these findings, we believe that cold plasma represents a promising new adjunct for cancer therapy, offering the ability to directly target and selectively kill neoplastic tissue. Notably, our plasma jet device provides a method for practical administration of this cancer therapy. Plasma therapy could potentially target internal malignancies via an endoscopic delivery system, thus enabling this technology to serve as either a stand-alone treatment option or, more realistically, as an adjuvant to existing therapies.

## Conclusions

In summary, this proof-of-principle study shows new *in vitro* and *in vivo* response of cancer cells upon treatment with cold plasma jets. These very surprising preliminary results suggest that the cold plasma jet can selectively ablate some cancer cells (such as melanoma and bladder), while leaving their corresponding normal cells essentially unaffected. The two best known cold plasma effects, plasma-induced apoptosis and the decrease of cell migration velocity (Fridman *et al*, 2008; Shashurin *et al*, 2008) can have important implications in cancer treatment by localising the affected area of the tissue and by decreasing metastasic development. Moreover, the selective effect of cold plasma on different cell types suggest that it is possible to find the right conditions with plasma treatment affecting only cancer cells, whereas leaving normal cells essentially unharmed. Finally, mid-sized tumours in nude mice were destroyed after a 2-min single-time treatment by cold plasma without thermal damage. As such, we expect that the development of cold plasma treatment will cause a paradigm shift in cancer therapy.
