# Supplementary material for: Cold plasma selectivity and the possibility of a paradigm shift in cancer therapy
Source: Br J Cancer. 2011 Oct 6;105(9):1295–301. doi: 10.1038/bjc.2011.386 (PMC3241555; doi:10.1038/bjc.2011.386)

Supplementary Table 1. Differentially expressed genes after plasma application

SYMBOL	Fold change	Regulation	Entrez Gene Name	Location
AADACL2	-5.2	down	arylacetamide deacetylase-like 2	unknown
ABCG4	-5.0	down	ATP-binding cassette, sub-family G (WHITE), member 4	Plasma Membrane
ABHD5	-5.2	down	abhydrolase domain containing 5	Cytoplasm
ACSBG1	-4.6	down	acyl-CoA synthetase bubblegum family member 1	Cytoplasm
ADAM8	42.9	up	ADAM metallopeptidase domain 8	Plasma Membrane
ADAMTS1	5.1	up	ADAM metallopeptidase with thrombospondin type 1 motif, 1	Extracellular Space
AFAP1L1	-4.5	down	actin filament associated protein 1-like 1	unknown
AIF1	5.4	up	allograft inflammatory factor 1	Nucleus
ALDH1A3	6.1	up	aldehyde dehydrogenase 1 family, member A3	Cytoplasm
ALDOA	4.8	up	aldolase A, fructose-bisphosphate	Cytoplasm
AMY1A	6.8	up	amylase, alpha 1A (salivary)	Extracellular Space
AMY1B	4.6	up	amylase, alpha 1B (salivary)	unknown
ANKRD37	6.8	up	ankyrin repeat domain 37	unknown
APCDD1	-8.7	down	adenomatosis polyposis coli down-regulated 1	unknown
APOBEC3B	4.0	up	apolipoprotein B mRNA editing enzyme, catalytic polypeptide-like 3B	Cytoplasm
ARG1	-12.7	down	arginase, liver	Cytoplasm
ARHGEF16	8.1	up	Rho guanine nucleotide exchange factor (GEF) 16	Cytoplasm
ARHGEF5L	-4.2	down	Rho guanine nucleotide exchange factor (GEF) 35	unknown
ASF1B	4.1	up	ASF1 anti-silencing function 1 homolog B (S. cerevisiae)	Nucleus
ASS1	4.5	up	argininosuccinate synthase 1	Cytoplasm
ATP2A1	-77.0	down	ATPase, Ca++ transporting, cardiac muscle, fast twitch 1	Cytoplasm
ATP6V1C1	-4.9	down	ATPase, H+ transporting, lysosomal 42kDa, V1 subunit C1	Cytoplasm
AVEN	-4.2	down	apoptosis, caspase activation inhibitor	Cytoplasm
B3GNT5	-4.4	down	UDP-GlcNAc:betaGal beta-1,3-N-acetylglucosaminyltransferase 5	unknown
BAG1	-4.0	down	BCL2-associated athanogene	Cytoplasm
BAHD1	21.7	up	bromo adjacent homology domain containing 1	unknown
BBOX1	4.3	up	butyrobetaine (gamma), 2-oxoglutarate dioxygenase	Cytoplasm
BCHE	44.0	up	butyrylcholinesterase	Plasma Membrane
BCL6	-4.5	down	B-cell CLL/lymphoma 6	Nucleus
BGN	5.8	up	biglycan	Extracellular Space
BST2	4.2	up	bone marrow stromal cell antigen 2	Plasma Membrane
BTN2A2	-29.8	down	butyrophilin, subfamily 2, member A2	unknown
C10orf10	7.8	up	chromosome 10 open reading frame 10	Cytoplasm

Supplementary Table 1. Differentially expressed genes after plasma application

SYMBOL	Fold change	Regulation	Entrez Gene Name	Location
C13orf15	-5.3	down	chromosome 13 open reading frame 15	Cytoplasm
C15orf48	5.8	up	chromosome 15 open reading frame 48	Nucleus
C19orf10	4.7	up	chromosome 19 open reading frame 10	Extracellular Space
C1orf110	52.6	up	chromosome 1 open reading frame 110	unknown
C2orf55	6.1	up	chromosome 2 open reading frame 55	unknown
C6orf15	-4.2	down	chromosome 6 open reading frame 15	Extracellular Space
C6orf188	-49.3	down	family with sequence similarity 26, member E	unknown
C9orf37	7.8	up	chromosome 9 open reading frame 37	unknown
C9orf41	-4.1	down	chromosome 9 open reading frame 41	unknown
CA6	-80.0	down	carbonic anhydrase VI	Extracellular Space
CADM1	-9.3	down	cell adhesion molecule 1	Plasma Membrane
CAMTA1	-29.6	down	calmodulin binding transcription activator 1	unknown
CCDC85B	4.0	up	coiled-coil domain containing 85B	unknown
CCDC90A	-4.5	down	coiled-coil domain containing 90A	unknown
CCDC99	6.4	up	coiled-coil domain containing 99	unknown
CCL27	-7.0	down	chemokine (C-C motif) ligand 27	Extracellular Space
CCNA1	-4.6	down	cyclin A1	Nucleus
CD74	227.8	up	CD74 molecule, major histocompatibility complex, class II invariant chain	Plasma Membrane
CDT1	6.7	up	chromatin licensing and DNA replication factor 1	Nucleus
CEACAM6	9.3	up	carcinoembryonic antigen-related cell adhesion molecule 6	Plasma Membrane
CENTD2	4.2	up	ArfGAP with RhoGAP domain, ankyrin repeat and PH domain 1	Cytoplasm
CFH	8.1	up	complement factor H	Extracellular Space
CGNL1	8.9	up	cingulin-like 1	unknown
CHPT1	-4.6	down	choline phosphotransferase 1	unknown
CHRNA9	-25.3	down	cholinergic receptor, nicotinic, alpha 9	Plasma Membrane
CLOCK	4.8	up	clock homolog (mouse)	Nucleus
CNTNAP2	-9.4	down	contactin associated protein-like 2	Plasma Membrane
COBL	5.0	up	cordon-bleu homolog (mouse)	unknown
CORO6	-4.9	down	coronin 6	unknown
CPVL	9.3	up	carboxypeptidase, vitellogenic-like	unknown
CSF2	7.9	up	colony stimulating factor 2 (granulocyte-macrophage)	Extracellular Space
CSNK2A2	-4.2	down	casein kinase 2, alpha prime polypeptide	Cytoplasm
CTXN1	5.0	up	cortexin 1	unknown

Supplementary Table 1. Differentially expressed genes after plasma application

SYMBOL	Fold change	Regulation	Entrez Gene Name	Location
CYBRD1	-8.2	down	cytochrome b reductase 1	Cytoplasm
CYFIP2	-66.7	down	cytoplasmic FMR1 interacting protein 2	Cytoplasm
CYR61	4.1	up	cysteine-rich, angiogenic inducer, 61	Extracellular Space
DAZ2	41.0	up	deleted in azoospermia 2	Cytoplasm
DAZ4	755.5	up	deleted in azoospermia 4	Cytoplasm
DBP	11.7	up	D site of albumin promoter (albumin D-box) binding protein	Nucleus
DCPS	4.6	up	decapping enzyme, scavenger	Nucleus
DEFB103B	5.8	up	defensin, beta 103B	Extracellular Space
DHRS9	4.8	up	dehydrogenase/reductase (SDR family) member 9	Cytoplasm
DMRT2	10.5	up	doublesex and mab-3 related transcription factor 2	Nucleus
DNER	-34.7	down	delta/notch-like EGF repeat containing	Plasma Membrane
DUSP1	-4.3	down	dual specificity phosphatase 1	Nucleus
EDG7	6.0	up	lysophosphatidic acid receptor 3	Plasma Membrane
EEF1A2	-29.0	down	eukaryotic translation elongation factor 1 alpha 2	Cytoplasm
FAM128A	27.9	up	mitotic spindle organizing protein 2A	unknown
FAM13A	-4.3	down	family with sequence similarity 13, member A	unknown
FAM181B	5.3	up	family with sequence similarity 181, member B	unknown
FETUB	-5.2	down	fetuin B	Extracellular Space
FKBP2	4.4	up	FK506 binding protein 2, 13kDa	Cytoplasm
FKBP5	-11.0	down	FK506 binding protein 5	Nucleus
FLG2	-7.1	down	filaggrin family member 2	unknown
FOXA2	21.9	up	forkhead box A2	Nucleus
GAA	4.6	up	glucosidase, alpha; acid	Cytoplasm
GBP6	6.1	up	guanylate binding protein family, member 6	unknown
GJC2	13.9	up	gap junction protein, gamma 2, 47kDa	Plasma Membrane
GLCCI1	-10.5	down	glucocorticoid induced transcript 1	unknown
GLRX	-8.5	down	glutaredoxin (thioltransferase)	Cytoplasm
GPX7	-114.9	down	glutathione peroxidase 7	Cytoplasm
GUCA2A	-16.9	down	guanylate cyclase activator 2A (guanylin)	Extracellular Space
HAL	-5.0	down	histidine ammonia-lyase	Cytoplasm
HAS3	5.5	up	hyaluronan synthase 3	Plasma Membrane
HBEGF	6.0	up	heparin-binding EGF-like growth factor	Extracellular Space
HCLS1	-113.5	down	hematopoietic cell-specific Lyn substrate 1	Nucleus

Supplementary Table 1. Differentially expressed genes after plasma application

SYMBOL	Fold change	Regulation	Entrez Gene Name	Location
HIST1H2AB	-5.7	down	histone cluster 1, H2ab	Nucleus
HIST1H2AE	-8.9	down	histone cluster 1, H2ae	Nucleus
HLA-DPA1	41.3	up	major histocompatibility complex, class II, DP alpha 1	Plasma Membrane
HLA-DQA1	135.9	up	major histocompatibility complex, class II, DQ alpha 1	Plasma Membrane
HLA-DRA	4.7	up	major histocompatibility complex, class II, DR alpha	Plasma Membrane
HLA-DRB1	12.6	up	major histocompatibility complex, class II, DR beta 1	Plasma Membrane
HLA-DRB6	18.8	up	major histocompatibility complex, class II, DR beta 6 (pseudogene)	unknown
HMGB3	-6.0	down	high-mobility group box 3	Nucleus
HNRNPF	-49.3	down	heterogeneous nuclear ribonucleoprotein F	Nucleus
HNRNPUL2	5.0	up	heterogeneous nuclear ribonucleoprotein U-like 2	Nucleus
HNRPLL	-4.0	down	heterogeneous nuclear ribonucleoprotein L-like	unknown
HOMER2	-5.6	down	homer homolog 2 (Drosophila)	Plasma Membrane
HPGD	-6.0	down	hydroxyprostaglandin dehydrogenase 15-(NAD)	Cytoplasm
HRNR	-8.2	down	hornerin	Cytoplasm
HSPA14	-4.1	down	heat shock 70kDa protein 14	Cytoplasm
HSPB3	-4.9	down	heat shock 27kDa protein 3	unknown
ICAM2	66.0	up	intercellular adhesion molecule 2	Plasma Membrane
IDI1	-4.7	down	isopentenyl-diphosphate delta isomerase 1	Cytoplasm
IER3	4.1	up	immediate early response 3	Cytoplasm
IGFBP2	5.7	up	insulin-like growth factor binding protein 2, 36kDa	Extracellular Space
IL1F9	16.4	up	interleukin 1 family, member 9	Extracellular Space
IL1R1	-49.4	down	interleukin 1 receptor, type I	Plasma Membrane
IL1RL1	32.2	up	interleukin 1 receptor-like 1	Plasma Membrane
IL8	12.9	up	interleukin 8	Extracellular Space
ITFG2	-4.3	down	integrin alpha FG-GAP repeat containing 2	unknown
ITPRIPL2	4.3	up	inositol 1,4,5-triphosphate receptor interacting protein-like 2	unknown
JMY	-5.0	down	junction mediating and regulatory protein, p53 cofactor	Nucleus
KCNQ1OT1	-5.5	down	KCNQ1 overlapping transcript 1 (non-protein coding)	unknown
KHDRBS3	5.6	up	KH domain containing, RNA binding, signal transduction associated 3	Nucleus
KPNA7	4.8	up	karyopherin alpha 7 (importin alpha 8)	unknown
KRT13	6.3	up	keratin 13	Cytoplasm
KRT2	110.5	up	keratin 2	Cytoplasm
KRT37	4.3	up	keratin 37	Cytoplasm

Supplementary Table 1. Differentially expressed genes after plasma application

SYMBOL	Fold change	Regulation	Entrez Gene Name	Location
KRTAP19-6	-5.0	down	keratin associated protein 19-6	unknown
KRTAP21-2	-19.2	down	keratin associated protein 21-2	unknown
KRTAP6-3	-18.5	down	keratin associated protein 6-3	unknown
KRTAP7-1	-5.7	down	keratin associated protein 7-1 (gene/pseudogene)	unknown
LCE2B	-6.7	down	late cornified envelope 2B	unknown
LCE2C	-8.6	down	late cornified envelope 2C	unknown
LCE2D	-9.7	down	late cornified envelope 2D	unknown
LCE6A	-4.4	down	late cornified envelope 6A	unknown
LCP1	15.7	up	lymphocyte cytosolic protein 1 (L-plastin)	Cytoplasm
LIPM	-5.1	down	lipase, family member M	Extracellular Space
LY6G6C	-4.4	down	lymphocyte antigen 6 complex, locus G6C	Extracellular Space
LYG1	-28.0	down	lysozyme G-like 1	Extracellular Space
LYPD1	4.8	up	LY6/PLAUR domain containing 1	Plasma Membrane
LYPD6B	-4.9	down	LY6/PLAUR domain containing 6B	unknown
MAGEA1	7.1	up	melanoma antigen family A, 1 (directs expression of antigen MZ2-E)	Plasma Membrane
MB	59.1	up	myoglobin	Cytoplasm
MIR1974	9.1	up	microRNA 1974	unknown
MIR574	-5.4	down	microRNA 574	unknown
MSMB	-30.4	down	microseminoprotein, beta-	Extracellular Space
MT1M	-6.8	down	metallothionein 1M	unknown
MT4	-7.0	down	metallothionein 4	unknown
MYL1	-6.6	down	myosin, light chain 1, alkali; skeletal, fast	Cytoplasm
NBPF20	4.4	up	neuroblastoma breakpoint family member	unknown
NDRG2	7.9	up	NDRG family member 2	Cytoplasm
NDST1	5.2	up	N-deacetylase/N-sulfotransferase (heparan glucosaminyl) 1	Cytoplasm
NEB	-6.9	down	nebulin	Cytoplasm
NHLH2	-4.3	down	nescient helix loop helix 2	Nucleus
NRTN	-32.7	down	neurturin	Extracellular Space
OGFRL1	-9.3	down	opioid growth factor receptor-like 1	unknown
OTUB2	-50.4	down	OTU domain, ubiquitin aldehyde binding 2	unknown
PADI3	45.6	up	peptidyl arginine deiminase, type III	Cytoplasm
PCDH21	-40.6	down	cadherin-related family member 1	Plasma Membrane
PHLDA1	5.6	up	pleckstrin homology-like domain, family A, member 1	Cytoplasm

Supplementary Table 1. Differentially expressed genes after plasma application

SYMBOL	Fold change	Regulation	Entrez Gene Name	Location
PIK3R1	-10.7	down	phosphoinositide-3-kinase, regulatory subunit 1 (alpha)	Cytoplasm
PLA2G4A	46.3	up	phospholipase A2, group IVA (cytosolic, calcium-dependent)	Cytoplasm
PLA2G4F	-6.0	down	phospholipase A2, group IVF	Cytoplasm
PLAG1	-4.5	down	pleiomorphic adenoma gene 1	Nucleus
POLD1	70.8	up	polymerase (DNA directed), delta 1, catalytic subunit 125kDa	Nucleus
PPAP2B	20.9	up	phosphatidic acid phosphatase type 2B	Plasma Membrane
PPDPF	15.6	up	pancreatic progenitor cell differentiation	unknown
PPFIA4	4.7	up	protein tyrosine phosphatase	Plasma Membrane
PRAME	29.0	up	preferentially expressed antigen in melanoma	Nucleus
PRPF18	-6.9	down	PRP18 pre-mRNA processing factor 18 homolog (S. cerevisiae)	Nucleus
PRR11	31.2	up	proline rich 11	unknown
PTGS2	7.5	up	prostaglandin-endoperoxide synthase 2	Cytoplasm
PTPRU	4.8	up	protein tyrosine phosphatase, receptor type, U	Plasma Membrane
PYROXD1	-4.5	down	pyridine nucleotide-disulphide oxidoreductase domain 1	unknown
RASD2	5.7	up	RASD family, member 2	Cytoplasm
RASIP1	-7.2	down	Ras interacting protein 1	Cytoplasm
RCOR2	4.4	up	REST corepressor 2	Nucleus
REL	-5.3	down	v-rel reticuloendotheliosis viral oncogene homolog (avian)	Nucleus
RETNLB	-4.2	down	resistin like beta	Extracellular Space
RIMS2	-40.0	down	regulating synaptic membrane exocytosis 2	Cytoplasm
RNF187	6.3	up	ring finger protein 187	unknown
ROBO1	-6.3	down	roundabout, axon guidance receptor, homolog 1 (Drosophila)	Plasma Membrane
ROCK1	-9.8	down	Rho-associated, coiled-coil containing protein kinase 1	Cytoplasm
RPIA	-4.4	down	ribose 5-phosphate isomerase A	Cytoplasm
RRAD	-7.2	down	Ras-related associated with diabetes	Cytoplasm
RUNX3	4.5	up	runt-related transcription factor 3	Nucleus
S100A12	4.8	up	S100 calcium binding protein A12	Cytoplasm
SAA1	-4.1	down	serum amyloid A1	Extracellular Space
SDSL	11.2	up	serine dehydratase-like	unknown
SEMA5A	5.2	up	sema domain, cytoplasmic domain, (semaphorin) 5A	Plasma Membrane
SEPP1	5.3	up	selenoprotein P, plasma, 1	Extracellular Space
SESN1	-4.8	down	sestrin 1	Nucleus
SEZ6L2	6.3	up	seizure related 6 homolog (mouse)-like 2	unknown

Supplementary Table 1. Differentially expressed genes after plasma application

SYMBOL	Fold change	Regulation	Entrez Gene Name	Location
SGPP2	-4.7	down	sphingosine-1-phosphate phosphatase 2	Cytoplasm
SH3PXD2B	27.3	up	SH3 and PX domains 2B	unknown
SIGIRR	8.4	up	single immunoglobulin and toll-interleukin 1 receptor (TIR) domain	Plasma Membrane
SIGLEC12	-4.9	down	sialic acid binding Ig-like lectin 12	Plasma Membrane
SLC25A17	-4.3	down	solute carrier family 25	Cytoplasm
SLC25A42	5.0	up	solute carrier family 25, member 42	unknown
SLC29A4	-41.6	down	solute carrier family 29 (nucleoside transporters), member 4	Plasma Membrane
SLC6A8	5.5	up	solute carrier family 6	Cytoplasm
SLC7A6	-4.6	down	solute carrier family 7	Plasma Membrane
SMPD3	-4.3	down	sphingomyelin phosphodiesterase 3	Cytoplasm
SNORD3A	4.1	up	small nucleolar RNA, C/D box 3A	unknown
SOX9	6.1	up	SRY (sex determining region Y)-box 9	Nucleus
SPINK6	4.7	up	serine peptidase inhibitor, Kazal type 6	Extracellular Space
SPRR2A	9.7	up	small proline-rich protein 2A	Cytoplasm
SPRR2B	4.9	up	small proline-rich protein 2B	Cytoplasm
SRGAP3	-4.0	down	SLIT-ROBO Rho GTPase activating protein 3	unknown
STC2	7.7	up	stanniocalcin 2	Extracellular Space
STMN3	4.8	up	stathmin-like 3	Nucleus
STRBP	-5.5	down	spermatid perinuclear RNA binding protein	Cytoplasm
STYXL1	5.4	up	serine/threonine/tyrosine interacting-like 1	unknown
TCN1	7.5	up	transcobalamin I (vitamin B12 binding protein, R binder family)	Cytoplasm
TFPI2	5.4	up	tissue factor pathway inhibitor 2	Extracellular Space
TGM3	6.3	up	transglutaminase 3	Cytoplasm
TINAGL1	11.2	up	tubulointerstitial nephritis antigen-like 1	Extracellular Space
TLCD1	-5.5	down	TLC domain containing 1	unknown
TMEM178	-39.2	down	transmembrane protein 178	unknown
TNFRSF12A	5.9	up	tumor necrosis factor receptor superfamily, member 12A	Plasma Membrane
TNNT1	14.9	up	troponin T type 1 (skeletal, slow)	unknown
TNNT2	-4.0	down	troponin T type 2 (cardiac)	Cytoplasm
TOX2	-4.5	down	TOX high mobility group box family member 2	Nucleus
TP53INP1	-4.3	down	tumor protein p53 inducible nuclear protein 1	Nucleus
TSC22D3	-17.0	down	TSC22 domain family, member 3	Nucleus
TSHZ2	-4.4	down	teashirt zinc finger homeobox 2	unknown

Supplementary Table 1. Differentially expressed genes after plasma application

SYMBOL	Fold change	Regulation	Entrez Gene Name	Location
TSPAN10	6.7	up	tetraspanin 10	unknown
TYMP	4.9	up	thymidine phosphorylase	Extracellular Space
UHRF1BP1L	-4.2	down	UHRF1 binding protein 1-like	unknown
USP12	-4.6	down	ubiquitin specific peptidase 12	Cytoplasm
USP19	-4.0	down	ubiquitin specific peptidase 19	Cytoplasm
VAMP5	-5.3	down	vesicle-associated membrane protein 5 (myobrevin)	Plasma Membrane
VOPP1	4.9	up	vesicular, overexpressed in cancer, prosurvival protein 1	Nucleus
VTCN1	11.9	up	V-set domain containing T cell activation inhibitor 1	Plasma Membrane
VWA1	4.2	up	von Willebrand factor A domain containing 1	Extracellular Space
WFDC12	-4.5	down	WAP four-disulfide core domain 12	Extracellular Space
WNT11	-24.2	down	wingless-type MMTV integration site family, member 11	Extracellular Space
ZFP37	-7.5	down	zinc finger protein 37 homolog (mouse)	Nucleus
ZNF259	-4.4	down	zinc finger protein 259	Nucleus
ZNF77	-58.7	down	zinc finger protein 77	Nucleus
ZSCAN16	-4.8	down	zinc finger and SCAN domain containing 16	Nucleus

Supplementary Table 2 - *Top network functions of differentially expressed genes according to Ingenuity Pathway Analysis.*

	**NETWORK FUNCTIONS**
1	Organismal Injury and Abnormalities, Reproductive System Disease, Cellular Development
2	Cell Signaling, Cellular Movement, Dermatological Diseases and Conditions
3	Cell Death, Inflammatory Response, Cardiovascular Disease
4	Dermatological Diseases and Conditions, Immunological Disease, Inflammatory Disease
5	Cancer, Reproductive System Disease, Cardiac Inflammation

Supplementary Figure 1. Network schematics of up-regulated cellular signaling pathways after cold plasma application.

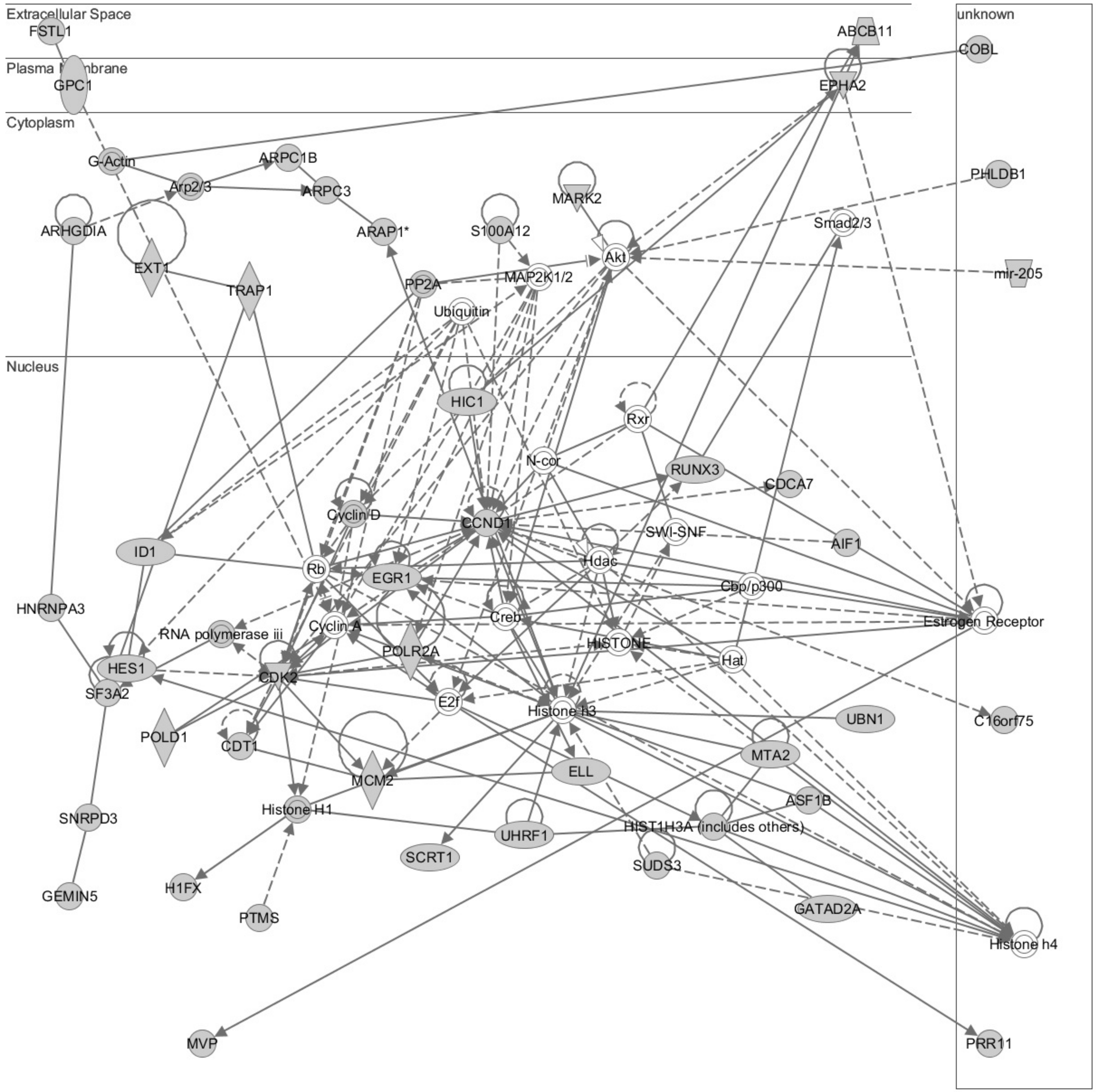

Supplementary Figure 2. Network schematics of down-regulated cellular signaling pathways after cold plasma application.

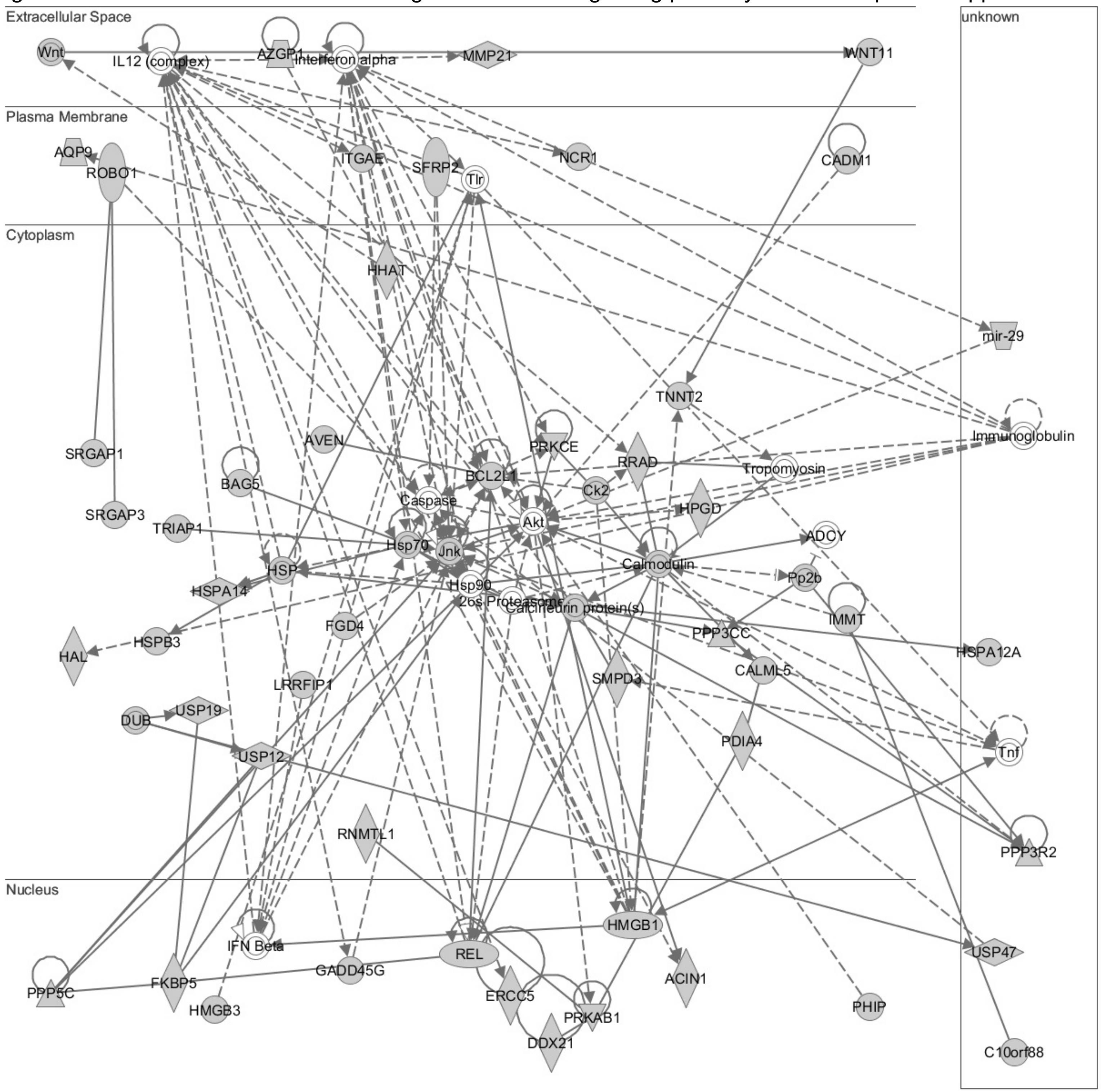

Supplement: Supplementary Information [file bjc2011386x1.pdf]
